# Next Generation Biobanking: Employing a Robotic System for Automated Mononuclear Cell Isolation

**DOI:** 10.1089/bio.2021.0181

**Published:** 2023-02-14

**Authors:** Yannick F. Fuchs, Jonathan Brunner, Marc Weigelt, Anja Schieferdecker, Robert Morgenstern, Andrea Sturm, Boris Winter, Helena Jambor, Friedrich Stölzel, Leo Ruhnke, Malte von Bonin, Elke Rücker-Braun, Falk Heidenreich, Anke Fuchs, Ezio Bonifacio, Martin Bornhäuser, David M. Poitz, Heidi Altmann

**Affiliations:** ^1^ Center for Regenerative Therapies Dresden, Technische Universität Dresden, Dresden, Germany.; ^2^ Medical Department I, University Hospital Carl Gustav Carus, Technische Universität Dresden, Dresden, Germany.; ^3^ National Center for Tumor Diseases (NCT), Partner Site Dresden, Germany and German Cancer Research Center (DKFZ), Heidelberg, Germany.; ^4^ Hamilton Bonaduz AG, Bonaduz, Switzerland.; ^5^ Mildred Scheel Early Career Center, Medical Faculty, Technische Universität Dresden, Dresden, Germany.; ^6^ Clinical Trials Unit, DKMS gGmbH, Dresden, Germany.; ^7^ Institute for Clinical Chemistry and Laboratory Medicine, University Hospital and Faculty of Medicine Carl Gustav Carus of TU Dresden, Dresden, Germany.

Biobanking of clinical samples is becoming increasingly important for studying human diseases and is essential for developing effective therapies. Peripheral blood mononuclear cells (PBMC), isolated at different times during disease and therapy, are fundamental for clinically relevant research.^1,2^ However, PBMC are manually isolated from blood with limited standardization of techniques among laboratories worldwide. The procedure requires a high expenditure in terms of the time required and the involvement of experienced personnel to ensure high operational capacity and to reduce pre-analytical variability.^3,4^ Despite the obvious need for a reproducible and automated system that is capable of isolating PBMC for biobanking, only a few pioneering works have been conducted to automate parts of this complex procedure.^5,6^

Here, we compiled, established, and validated a unique robotic system and an automated process that comprises all of the steps required to isolate PBMC, starting with the extraction of cells from the original blood collection tube through to counting and aliquoting the PBMC into cryovials for long-term storage.

We sought to develop an automated process that (1) is built on a classical manual density gradient workflow, (2) maintains the quality and quantity of PBMC, and (3) offers advantages in terms of consistency, hands-on time, throughput, sample tracking, and error reduction.

Our robotic system is based on five components (Fig. 1A), comprising a Hamilton Microlab STAR Autoload with four 1000-μL and four 5mL pipetting channels equipped with a Tube Twister and barcode scanner, a Hamilton LabElite Integrated I.D. Capper, and a Hettich Rotanta 460 Robotic centrifuge integrated with a Hamilton HMotion plate handler serving as the loading device. All components are enclosed and equipped with high-efficiency particulate air (HEPA) filter hoods and ultraviolet (UV) light options to reduce the risk of sample contamination.

**FIG. 1.**
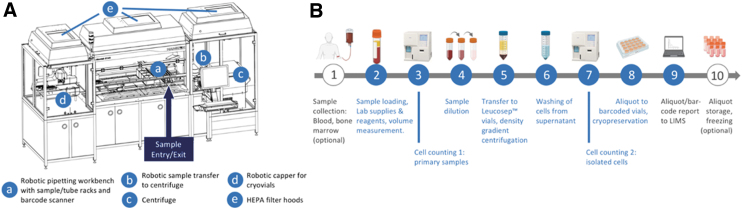
Schematics of the robotic system and workflow of automated PBMC processing. **(A)** Schematic of the mononuclear cell isolation system for automated PBMC processing. **(B)** Overview of the workflow used for the automated isolation of PBMC. Icons: Biorender. HEPA, high-efficiency particulate air; LIMS, laboratory information management system; PBMC, peripheral blood mononuclear cells.

With a processing time of 2–3.5 hours and a hands-on time of <15 minutes, up to 12 barcoded primary standard blood collection tubes (e.g., Sarstedt S-Monovette^®^ or BD Vacutainer^®^ for volumes between 2.7 and 10 mL) are processed in parallel via the workflow described below and presented in Figure 1B (Supplementary Table S1 provides further details regarding the individual processing steps).

First, the collection tubes are loaded and registered in the laboratory information management system (LIMS). The caps are removed, and the aliquots are taken for automated cell counting on a Sysmex XN-1000. The samples are then transferred into 50-mL tubes and diluted with phosphate-buffered saline (PBS). Leucosep™ tubes (50, or 12 mL for small blood samples) prefilled with separation medium are loaded with the diluted samples and transferred to the centrifuge. The supernatants containing the PBMC are then washed twice with PBS in new 50 mL tubes by centrifugation, and the supernatant is removed. The remaining PBMC pellets are resuspended in PBS, and the absolute cell numbers in each isolate are determined using a small aliquot.

After a final centrifugation step, the PBMC are resuspended in pre-cooled cryomedium and transferred into two-dimensional-barcoded cryovials. Automated de- and re-capping of vials facilitates this processing step. Samples are tracked throughout the process, and the final aliquot barcodes are reported back to the LIMS in preparation for the freezing process.

For validation, we compared manual and robotic blood cell purification using peripheral blood samples collected from healthy donors (approved by the Ethics Committee of TU Dresden). Samples were equally distributed to processing tubes and run in parallel by the robotic system and two experienced laboratory technicians using identical density gradients and centrifuge settings. The cell numbers and frequencies were determined using a Sysmex XN-1000 in whole blood before (pre) and after (post) PBMC purification to compare manual and robotic purification, recovery, and depletion of major cell types.

Within the leukocyte fraction, there was a strong reduction of neutrophils after purification (Fig. 2A) (median [interquartile range, IQR]; pre-purification: 53.7% [7.6%]; manual (post): 4.1% [2.0%]; robotic (post): 4.8% [2.7%]), which was accompanied by enrichment of the lymphocyte and monocyte fractions. Both purification methods provided high recovery of lymphocytes (manual: 75.5% [12.0%]; robotic: 91.8% [5.2%]; *p* < 0.0001) and monocytes (manual: 69.9% [13.9%]; robotic: 80.9% [9.2%]; *p* = 0.0002), although higher with robotic purification (Fig. 2B).

**FIG. 2.**
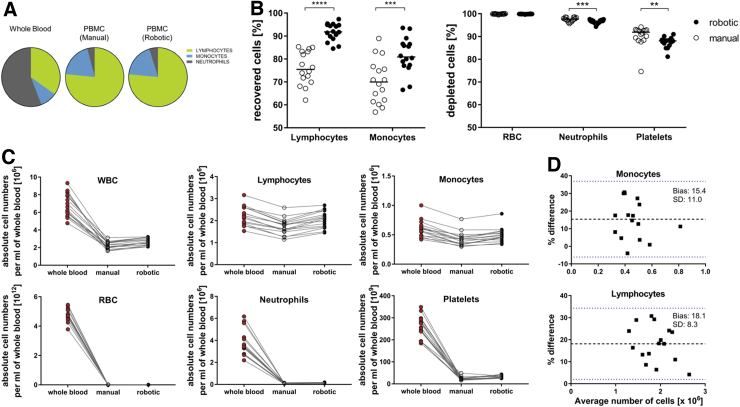
Comparison of manual and robotic PBMC isolation. **(A)** Pie charts showing the mean frequencies of the main leukocyte populations in whole blood and isolated PBMC obtained from 16 healthy donors. **(B)** Scatter plots showing the frequencies of cell populations recovered (*left*) or depleted (*right*) from whole blood during PBMC purification. The individual points represent the median values of duplicates processed by either two experienced technicians (manual) or the robotic system. The *lines* indicate the median values of all 16 samples per group. **(C)** Absolute cell numbers of the indicated cell types in whole blood (*red circles*) or among PBMC obtained by manual (*open circles*) or robotic (*black circles*) processing. Cell numbers were counted using an automated cell analyzer. The individual points represent the median of 5 (pre-processing of whole blood samples) or 2 replicates (after manual or robotic processing) for all 16 donor samples. The *lines* connect the data points for the same donor. **(D)** Bland–Altman plots for comparison of manual and robotic processing of lymphocytes and monocytes. **p < 0.01, ***p < 0.001, and ****p < 0.0001 (Wilcoxon matched-pairs signed-rank test). RBC, red blood cells; SD, standard deviation.

This also led to increased absolute cell numbers for the robotic process (Fig. 2C); the bias is shown in the Bland–Altmann diagram (Fig. 2D). The depletion efficiencies for red blood cells (RBC; manual: 99.9% [0.1%]; robotic: 99.9% [0.02]; *p* = n.s.), neutrophils (manual: 97.7% [1.3%]; robotic: 96.8% [0.9%]; *p* = 0.0002) and platelets (91.9% [3.6%] robotic: 88.1% [1.7%]; *p* = 0.0063) were also high using both strategies (Fig. 2B). In addition, and in accordance with the advantages of automation in biobanking (i.e., standardization, error reduction, and reproducibility^7^), our robotic workflow shows minimal variation for lymphocyte retrieval in sample replicates (median %coefficient of variation [IQR]; manual: 4.63 [4.57]; robotic: 1.80 [1.98]) (Supplementary Fig. S1).

To assess the quality and composition of the purified cells, we adopted and used previously described flow cytometry panels^8^ to analyze a total of 30 immune subsets comprising 4 monocyte, 20 lymphocyte, and 6 dendritic cell subsets among PBMC isolated from whole blood donation products obtained from the local blood donation service. The viability of the CD45^+^ leukocyte population was high for both processes (median [IQR]; manual: 96.2% [4.6%]; robotic: 96.2% [4.1%]) (Fig. 3A and Supplementary Fig. S2 for flow cytometry gating strategies).

**FIG. 3.**
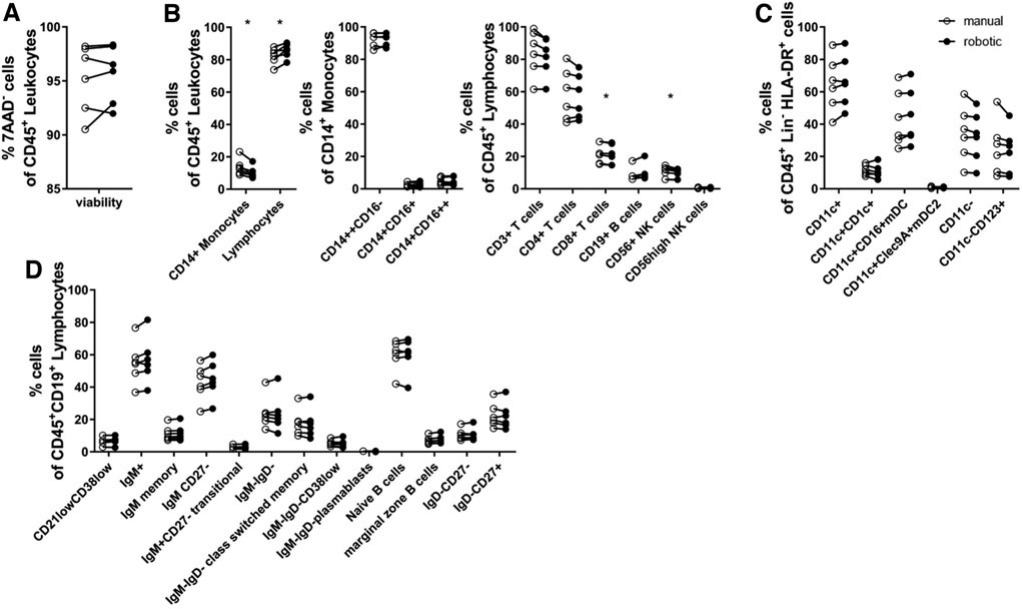
Flow cytometric comparison of PBMC isolated using the manual and robotic processing methods. **(A)** Dot plot comparing the frequencies of viable (7AAD-negative) CD45^+^ leukocytes after manual and robotic purification of PBMC from whole blood samples from six donors. Cell frequencies were determined by flow cytometry. **(B–D)** Dot plots of multicolor flow cytometry analyses of the purified PBMC from the same donors. Frequencies of representative cell populations within leukocytes, including monocytes and lymphocytes **(B)**, dendritic cells **(C)**, and B cells **(D)**. *p < 0.05 (Wilcoxon matched-pairs signed-rank test). 7AAD, 7-aminoactinomycin D; HLA, human leukocyte antigen; Ig, immunoglobulin; NK, natural killer.

In contrast to PBMC purified from fresh blood (<2 hours from blood draw) (Fig. 2B), whole blood donations yielded higher lymphocyte and lower monocyte percentages after robotic versus manual sample processing (Fig. 3B), resulting in an elevated lymphocyte-to-monocyte ratio (manual: 6.4 [4.0]; robotic: 9.8 [5.2], *p* = 0.03). Within the monocyte and the lymphocyte populations, there were no striking differences in the proportions of subpopulations between the two processing methods (Fig. 3B–D). Thus, all expected immune-cell subsets were detected and no major differences in subpopulation frequencies were observed between manual and robotic sample processing.

In summary, we have developed a novel robotic system for automated processing of PBMC from whole blood. Compared with manual processing, the robotic system achieved equivalent cell yield, composition, and viability according to the results of Sysmex and multi-parameter flow cytometry.

Biobanking and standardization via workflow automation has become increasingly important for improving the quality of biological samples.^9–11^ However, until now, very few pioneering studies have focused on the partial automation of PBMC isolation.^5,6^ Our system involves collecting the PBMC-containing supernatant from Leucosep tubes after centrifugation by pipetting the sample located above the porous barrier in the tube while minimizing recontamination with separation media.

Capacitive liquid level detection is performed to sense the level of liquid within the sample and combined with the predefined minimum Z-height of the pipette during this step to guide buffy coat and plasma collection. In addition, an experimentally tested, programmed algorithm using hematocrit data from the initial cell count step is used to select whether a 12 or 50 mL Leucosep tube is necessary to ensure the PBMC are located above the porous barrier after density centrifugation.

The system also integrates several important cell isolation steps into the automated workflow, namely blood sample registration and cryovial identification via barcode scanning, automated cell counting for decision-making during sample processing, transferring the samples to a centrifuge and performing centrifugation, uploading the entire data handling process to the LIMS, and finally cell yield-based filling of cryovials with cryovial de- and re-capping. All of these steps reduce the hands-on time and allow strategic optimization and standardization of the entire process.

The robotic system is equipped with HEPA filter hoods and UV light for decontamination between runs. In addition, all pipetting steps, except for distribution of sterile PBS, are performed in the “surface mode,” which means the pipetting tip contacts the liquid throughout dispensing and mixing. During dispensation and mixing, the pipetting precisely follows the liquid level in the tube to stay submerged. This is expected to reduce the risk of aerosol formation.

The robotic system has a four-channel setup. The minimum processing time of 2 hours is achieved when up to four samples are processed simultaneously. The duration of time that the cells are in contact with either the separation media or with cryomedia is likely to influence the yield and quality of isolated cells, and this prompted us to develop a workflow designed to minimize this time. We think this is important when processing 5–12 samples in an interlocked manner, with a processing time of 2.75 hours for 5–8 samples and 3.5 hours for 9–12 samples.

Based on our experience, the overall processing times are comparable to that of manual processes, and the robotic system is more favorable if more than six samples are processed per run, particularly because the robotic system automatically integrates the sample cell counting information and uses it to adjust the volume of cryomedia and to determine the number of cryovials to be filled, and performs parallel documentation of all relevant processing parameters according to Sample PREanalytical Code (SPREC) guidelines. Regardless of the number of samples, the robotic system greatly reduces the hands-on time while ensuring a high level of standardization and flexibility. It also allows the laboratory to process 12 samples with different volumes and from different primary collection tubes.

Although we observed a modest increase in the absolute number of PBMC recovered from fresh blood samples by our robotic system as compared with the parallel manual process, others have described a modest reduction or equal recovery of PBMC using their automated isolation systems.^5,6^ Collectively, these studies indicate that the automated systems will not dramatically affect cell recovery as compared with expert manual isolation.

Laboratory automation requires space (2044 × 3677 × 1124 mm [height × width × depth]) and is associated with high costs, particularly for purchasing and maintaining the equipment. However, the standardization of critical key methodology is important, and biobanks rely on standardized automation processes, although with a disadvantage of higher cost, at least initially. Currently, the costs of consumables for manual or robotic PBMC isolation are similar. Nevertheless, the use of defined consumables in standardized processes may provide an opportunity to establish recycling schemes.

In conclusion, our automatic robotic system can become a tool for collecting standardized, high-quality material for biobanking in the field of immunology and hematology that may advance research and novel approaches that are pertinent to disease prevention, diagnosis, and therapy.

## Supplementary Material

Supplemental data

Supplemental data

Supplemental data
