# Supplementary material for: Next Generation Biobanking: Employing a Robotic System for Automated Mononuclear Cell Isolation
Source: Biopreserv Biobank. 2023 Feb 14;21(1):106–10. doi: 10.1089/bio.2021.0181 (PMC9963478; doi:10.1089/bio.2021.0181)
Supplement: Supplemental data [file Suppl_TableS1.docx]

**Table S1: Overview on individual processing steps and status of automatization**

* the robotic system detects the sample volume via capacitive liquid level detection

**this step is currently manual but can be integrated and fully automated

*** for robotic PBMC isolation data are transferred throughout the entire process
