# Supplementary material for: Next Generation Biobanking: Employing a Robotic System for Automated Mononuclear Cell Isolation
Source: Biopreserv Biobank. 2023 Feb 14;21(1):106–10. doi: 10.1089/bio.2021.0181 (PMC9963478; doi:10.1089/bio.2021.0181)
Supplement: Supplemental data [file Suppl_FigS1.docx]

**Figure S1
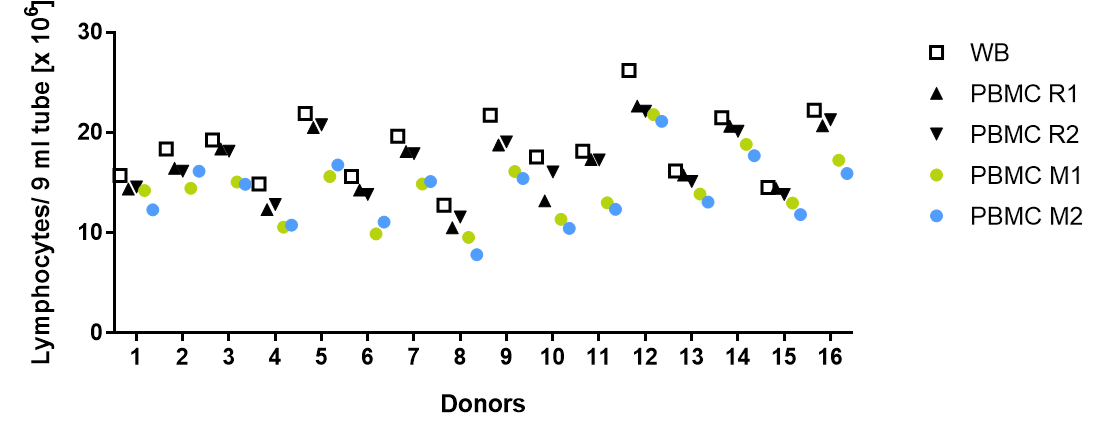
**

**Supplementary Figure S1:** **Lymphocyte numbers in whole blood and PBMC isolated via robotic or manual processing.** Individual data points for lymphocyte retrieval in PBMC isolated from 9 ml whole blood (WB) sample tubes using robotic (duplicates R1 and R2) or manual (duplicates M1 and M2) processing are depicted. Numbers of lymphocytes measured in WB are shown as a reference and represent median values replicates (n=5) for each of the 16 donors.
