# Supplementary material for: Next Generation Biobanking: Employing a Robotic System for Automated Mononuclear Cell Isolation
Source: Biopreserv Biobank. 2023 Feb 14;21(1):106–10. doi: 10.1089/bio.2021.0181 (PMC9963478; doi:10.1089/bio.2021.0181)
Supplement: Supplemental data [file Suppl_FigS2.docx]

**Figure S2**

**
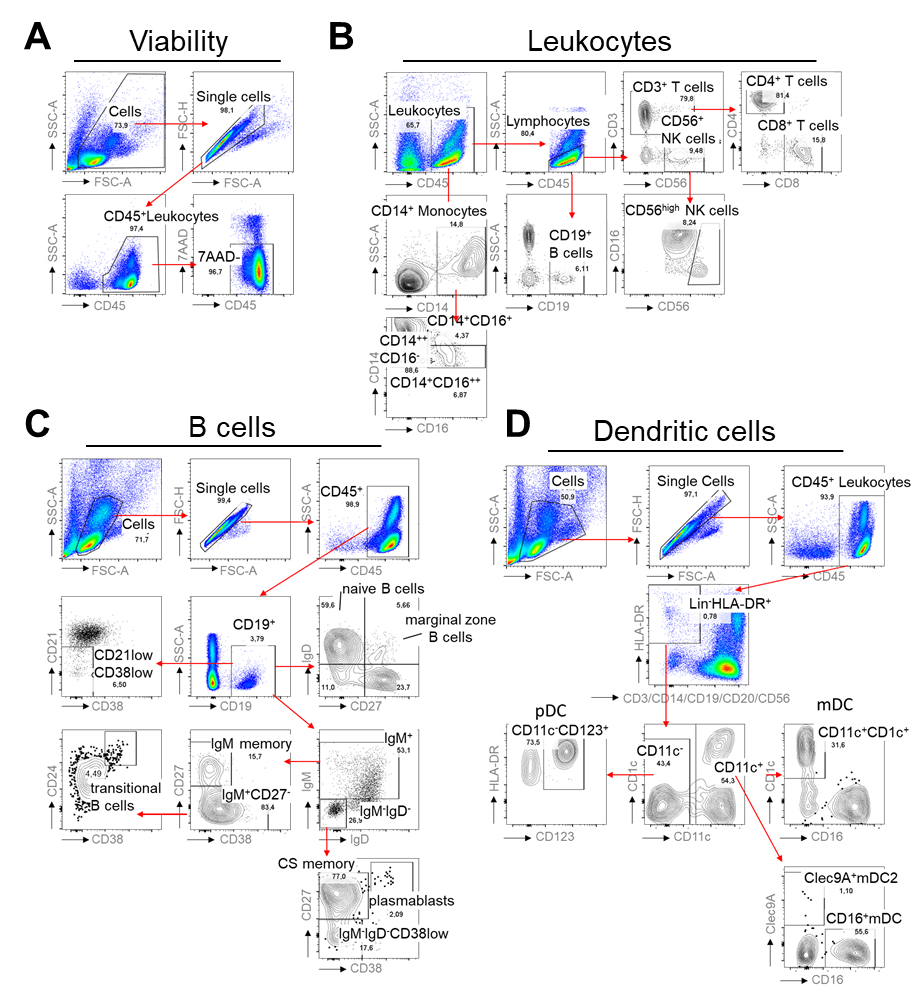
**

**Supplementary Figure S2:** **Flow cytometry gating strategies.** Strategies to determine the frequency of viable cells (**A**), major leukocyte populations (**B**), B cell subtypes (**C**), and dendritic cell populations (**D**) are exemplarily shown.
